# A General Rate-Distortion Optimization Method for Block Compressed Sensing of Images

**DOI:** 10.3390/e23101354

**Published:** 2021-10-16

**Authors:** Qunlin Chen, Derong Chen, Jiulu Gong

**Affiliations:** School of Mechatronical Engineering, Beijing Institute of Technology, Beijing 100081, China; 3120170115@bit.edu.cn (Q.C.); cdrmy@263.net (D.C.)

**Keywords:** data acquisition, compressed sensing, rate-distortion, optimal bit-depth, bit-rate, quantization

## Abstract

Block compressed sensing (BCS) is a promising technology for image sampling and compression for resource-constrained applications, but it needs to balance the sampling rate and quantization bit-depth for a bit-rate constraint. In this paper, we summarize the commonly used CS quantization frameworks into a unified framework, and a new bit-rate model and a model of the optimal bit-depth are proposed for the unified CS framework. The proposed bit-rate model reveals the relationship between the bit-rate, sampling rate, and bit-depth based on the information entropy of generalized Gaussian distribution. The optimal bit-depth model can predict the optimal bit-depth of CS measurements at a given bit-rate. Then, we propose a general algorithm for choosing sampling rate and bit-depth based on the proposed models. Experimental results show that the proposed algorithm achieves near-optimal rate-distortion performance for the uniform quantization framework and predictive quantization framework in BCS.

## 1. Introduction

Compressed sensing (CS) is a signal acquisition framework [1,2,3] that acquires the signal’s measurements by linear projection at the sub-Nyquist rate. Unlike traditional image coding methods with high computational complexity, the CS-based image coding methods are suitable for resource-constrained application scenarios through simultaneous data acquisition and compression [4,5,6,7].

When CS is applied to an image, the large measurement matrix will cause enormous computation and memory space for the codec. Gan [8] has proposed a block compressed sensing (BCS) method to decrease the measurement matrix’s size for images. BCS uses the same measurement matrix to measure the image block’s raster scan vector, significantly reducing the sensor’s calculation and transmission cost [9]. BCS processes each image block independently and supports parallel encoding, which can quickly obtain the image measurements. However, the real-valued CS measurements need to be combined with quantization and entropy encoder to output bitstreams for transmission or storage [10].

Although the uniform scalar quantization (SQ) is the most straightforward solution for quantizing CS measurements, it is inefficient in rate-distortion performance [6,11]. Therefore, some researchers have proposed different quantization schemes of CS measurements to enhance the rate-distortion performance. For example, Mun et al. [12] have combined the differential pulse-code modulation (DPCM) with uniform scalar quantization (DPCM-plus-SQ) for BCS measurements. The CS-based imaging system with DPCM-plus-SQ and the smoothed projected Landweber (SPL) reconstruction can compete with JPEG in some cases. Wang et al. [13] have proposed a progressive quantization framework of CS measurements, which is slightly better than JPEG in rate-distortion performance. Chen et al. [14] have proposed a progressive non-uniform quantization framework of CS measurements using partial Hadamard matrix together with patch-based recovery algorithm, which can reach the rate-distortion performance of CCSDS-IDC (consultative committee for space data systems-image data compression standard). Chen et al. [15] have proposed a multi-layer residual coding framework for CS measurements, which combines prediction with the uniform SQ at the encoder. The framework predicts the CS measurements by using the reconstructed image of the encoded CS measurements and then uses the uniform SQ to quantify the residuals between the predicted measurement and the actual measurement, which can obtain a better rate-distortion performance than JPEG2000. Some other quantization schemes are also used for CS measurements [16,17,18,19], but they are rarely used for CS-based image coding because of their complexity.

In a CS-based image coding system, the bit-rate and reconstruction distortion depend on the CS sampling rate and quantization bit-depth, which have competition at a given bit-budget [20]. Therefore, the encoder needs to assign a CS sampling rate and a bit-depth by rate-distortion optimization (RDO).

Some researchers have discussed the optimization problem of CS sampling rate and bit-depth. Chen et al. [21] have proposed a bit-rate model and a relative distortion model to assign CS sampling rate and bit-depth for the CS-based coding system with uniform SQ. Jiang et al. [22] have presented a new Lagrange multiplier method to set quantization step size and number of measurements, whereas they do not consider the complexity. Liu et al. [23] have introduced a distortion model of compressed video sampling to optimize the sampling rate and bit-depth. The model’s parameters need to be predicted from video features, which is not suitable for images. However, the above works only apply to uniform SQ scheme. To the knowledge of authors, little attention has been paid to optimize the CS sampling rate and bit-depth for other quantization schemes in BCS.

The purpose of this study is to propose an efficient RDO algorithm to assign the CS sampling rate and bit-depth for the most frequently used quantization schemes in BCS. The RDO algorithm should be designed with low complexity due to the simple coding process of CS. In this paper, we propose a bit-rate model and an optimal bit-depth model to avoid the high complexity of calculating rate-distortion cost. Firstly, we use generalized Gaussian distribution to describe the distribution of objects encoded by entropy encoder and then build a bit-rate model. Secondly, we find that there is a logarithmic relationship between the optimal quantized bit-depth and the bit-rate. Then, we propose an optimal bit-depth model and use a feed-forward neural network to train the model parameters. Finally, we introduce a general method for optimizing the CS sampling rate and bit-depth with the proposed models.

The remainder of this paper is structured as follows. We describe the problem of RDO in a CS-based imaging system in Section 2. Section 3 and Section 4 discuss the proposed bit-rate model and the optimal bit-depth model. We propose an algorithm to assign CS sampling rate and bit-depth in Section 5. The experiment results and conclusions are drawn in Section 6 and Section 7.

## 2. Problem Statement

CS theory states that a sparse signal can be recovered through its measurements obtained by linear random projection. Many natural images have a sparse representation in a wavelet transform domain or discrete cosine transform domain [24,25], so they can be acquired by CS. Suppose $x\in {\mathbb{R}}^{N\times 1}$ denotes a raster-scanned vector of an image block. The CS measurements vector $y\in {\mathbb{R}}^{M\times 1}$ of x can be acquired by the following expression:

(1) y=Φx,

where $\Phi \in {\mathbb{R}}^{M\times N}(M\ll N)$ is a measurement matrix, and the sampling rate or measurement rate is m=M/N.

Since CS measurements are real, they need to be discretized by the quantization before entropy encoding. Based on the most commonly used quantization schemes of CS measurements, the CS sampling model with quantization can be unified into the following expression:

(2) $$y_{Q}=Q_{b}\left[f\left(y\right)\right]=Q_{b}\left[f\left(\Phi x\right)\right],$$

where $y_{Q}$ is the quantized measurement vector, and it also stands for the input of entropy encoder. $Q_{b}:\mathbb{R}\to \mathbb{Q}$ denotes a uniform SQ operation for b-bit (applied element-wise in (2)), which maps f(y) to the discrete alphabet ℚ with $\left|\mathbb{Q}\right|=2^{b}$. In this paper, we define $Q_{b}\left[f\left(y\right)\right]=\frac{f\left(y\right)}{\Delta }$, where $\Delta =\frac{f_{\max}\left(y\right)-f_{\min}\left(y\right)}{2^{b}}$ is the uniform quantization step size, $f_{\max}\left(y\right)$ and $f_{\min}\left(y\right)$ represent the maximum and minimum of f(y), respectively. f(·) represents a reversible transform, which is used to change the distribution type of y. When the CS measurements are quantized by uniform SQ, f(·) is an identity transformation. When the CS measurements are quantized by A-law or μ-Law non-uniform quantization, f(·) is the law function [26]. When the CS measurements are quantized by prediction with uniform SQ, f(·) is the prediction function [12,17]. For example, in the DPCM-plus-SQ framework, $f(y^{\left(j\right)})=y^{\left(j+1\right)}-y^{\left(j\right)}$, where $y^{\left(j\right)}$ represents the measurement vector of the *j*-th image block. The progressive quantization methods [13,14] are also prediction frameworks combined with uniform SQ. In the progressive quantization method, the CS measurements are divided into a basic layer and refinement layer for transmission after uniform SQ quantization with B bit. In the basic layer, all B significant bits of the quantization indexes are transmitted, so the prediction function is equivalent to the identity transformation. In the refinement layer, the least B_1_ < B significant bits of the quantization index are transmitted, so the dropped highest B-B_1_ bit is equivalent to the predicted value, and the retained B_1_ least significant bits are equivalent to the prediction residual.

**Figure 1:**
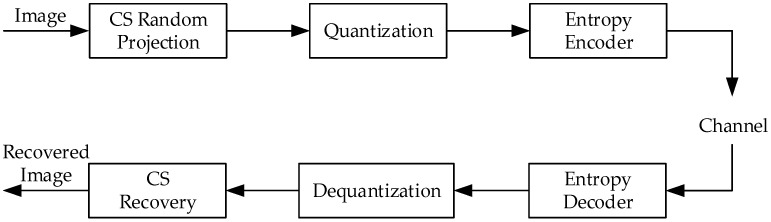
CS-based imaging system.

The CS-based image coding system is composed of CS sampling, quantization, and entropy encoder [15]. The bitstream of the encoded image is used for transmission or storage. The decoder restores the bitstream to an image through the corresponding entropy decoder, dequantization, and CS reconstruction algorithm. Figure 1 shows the flow chart of the CS-based imaging system [10].

The average number of bits per pixel [21] of the encoded image can be calculated by the following expression:

(3) R=mL,

where L is the average codeword length of the quantized CS measurements $y_{Q}$ after entropy encoding.

There is a positive correlation between average codeword length and quantization bit-depth. When the bit-rate is constrained, sampling rate and quantization bit-depth have a competitive relationship with each other. We can minimize the distortion to optimize the sampling rate and bit-depth for a given bit-rate $R_{goal}$, i.e.,

(4) $$\underset{m,b}{\arg\min}\;D(m,b,X)\;\;s.t.\;\;R(m,b,X)\leq R_{goal},$$

where R(m,b,X) and D(m,b,X), respectively, represent bit-rate and distortion of the image X at the sampling rate m and the bit-depth b. The bit-rate R(m,b,X) is the average number of bits per pixel of the encoded image, which can be obtained according to (3). Distortion refers to the dissimilarity between the reconstructed image $\hat{X}$ and the original image X. The distortion measures mainly include the mean square error (MSE), the peak signal-to-noise ratio (PSNR), and the structural similarity index measure (SSIM) [27]. The PSNR between the reconstructed image $\hat{X}$ and the original image X is used as a measure of distortion in our paper. The mathematical definition of PSNR is $\mathrm{PSNR}=10\times \log_{10}\left(255^{2}/\mathrm{MSE}(X,\hat{X})\right)$, where $\mathrm{MSE}(X,\hat{X})$ is the mean square error between the reconstructed image $\hat{X}$ and the original image X. The calculation of distortion and bit rate depends on the original image and decoded image, and the cost of obtaining decoded image is very expensive.

**Figure 2:**
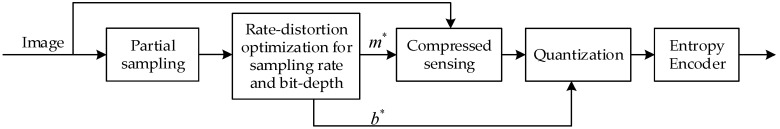
The CS-based coding system with RDO.

To avoid calculating the bit-rates and distortions, we first propose a new bit-rate model and an optimal bit-depth model. Then, we propose a general method to optimize the sampling rate and bit-depth for CS-based image coding. Figure 2 is the CS-based encoding system with RDO [21,23]. Our CS framework contains two CS processes. The first one is partial sampling, which aims to extract image features by a few CS measurements for RDO. The second one is to increase the number of CS measurements to achieve optimal sampling and compression by using the sampling rate optimized by RDO.

## 3. Bit-Rate Model

Based on (3), the bit-rate R depends on the average codeword length of the quantized CS measurements $y_{Q}$ after entropy encoding. The average codeword length can be approximated by information entropy of $y_{Q}$ before entropy encoding [28]. The information entropy is closely related to the distribution characteristics of the CS measurements, so we extract the distribution characteristics from the CS measurements of the first sampling to estimate the information entropy. However, the information entropy is only the lower boundary of the average codeword length. There is an error between the average codeword length and the information entropy estimated by a few measurements. Therefore, we modify the coefficients of the information entropy estimation model by fitting the offline data of the average codeword length and then take it as the average codeword length model.

### 3.1. Generalized Gaussian Distribution Model of the Quantized CS Measurements

According to (2), the quantized CS measurements can be considered to be obtained by f(·) and $Q_{b}\left[\cdot \right]$. $Q_{b}\left[\cdot \right]$ does not change the distribution type, while f(·) determines how to change the distribution type of CS measurements. The CS measurements using random Gaussian matrix obey Gaussian distribution [13]. When the structurally random matrix (SRM) is used for CS measurement, the CS measurements corresponding to the first row of SRM are uniformly distributed, and the remaining CS measurements are Laplacian distributed with zero mean [10]. The distribution of DPCM predictive errors without conditioning on contexts is very close to a Laplace distribution [29]. The experiments of [30] show that the prediction errors of DPCM-plus-SQ satisfy the generalized Gaussian distribution. The Gauss distribution, the uniform distribution, and the Laplace distribution belong to the generalized Gauss distribution with specific shape parameters. In order to describe the distribution of CS measurements more generality, we use the generalized Gauss distribution to describe f(y) and $y_{Q}$. The generalized Gaussian distribution density function with zero mean can be expressed as:

(5) $$p_{x}(x|v,\sigma )=C\left(\beta ,\sigma \right)\exp\left\{-{\left[\alpha \left(\beta ,\sigma \right)|x|\right]}^{\beta }\right\},$$

where $\alpha \left(\sigma ,\beta \right)=\sigma_{}^{-1}{\left[\frac{\Gamma (3/\beta )}{\Gamma (1/\beta )}\right]}^{\frac{1}{2}}$, $C\left(\sigma ,\beta \right)=\frac{\beta \alpha \left(\sigma ,\beta \right)}{2\Gamma (1/\beta )}$. σ is the standard deviation. β is the shape parameter, which determines the attenuation rate of the density function. $\Gamma (t)=\int_{0}^{\infty }e^{-u}u^{t-1}du$. The Laplace distribution and Gaussian distribution correspond to generalized Gaussian distribution when β=1 and β=2, respectively. Based on the generalized Gaussian distribution, the information entropy [31] of f(y) can be estimated as

(6) $$\begin{array}{l} H\;\approx -\int_{-\infty }^{+\infty }p_{f}(x|\beta ,\sigma )\log_{2}\left(p_{f}(x|\beta ,\sigma )\right)dx\\ \;\;\;\;=-\log_{2}\left[\frac{\beta \alpha (\beta ,\sigma_{f})}{2\Gamma (1/\beta )}\right]+\frac{1}{\beta \ln2}\\ \;\;\;\;=\log_{2}\left(2\sigma_{f}\right)-\log_{2}\left[\frac{\beta {\left[\Gamma (3/\beta )\right]}^{\frac{1}{2}}}{\Gamma {\left[\left(1/\beta \right)\right]}^{\frac{3}{2}}}\right]+\frac{1}{\beta \ln2} \end{array}$$

where $\sigma_{f}$ and β are the standard deviation and distribution shape parameters of f(y), respectively. In Equation (2), $y_{Q}$ is the discretization of f(y) quantized by the quantization step size Δ, so the information entropy [31] of $y_{Q}$ can be estimated by:

(7) $$\begin{array}{l} H_{Q}\;≃H-\log_{2}\Delta \\ =\log_{2}\left(2\sigma_{f}\right)-\log_{2}\left[\frac{\beta {\left[\Gamma (3/\beta )\right]}^{\frac{1}{2}}}{\Gamma {\left[\left(1/\beta \right)\right]}^{\frac{3}{2}}}\right]+\frac{1}{\beta \ln2}-\log_{2}\Delta \end{array}$$

### 3.2. Average Codeword Length Estimation Model

In Equation (7), $\sigma_{f}$ and β are keys to estimating information entropy H. However, $\sigma_{f}$ and β cannot be calculated directly because the CS measurements are unknown before the sampling rate and bit-depth are assigned. Since the number of CS measurements required for a high-quality reconstructed signal must satisfy a lower limit, the number of CS measurements used for compression will exceed the lower limit regardless of the goal bit-rate. Therefore, we can acquire a small number of CS measurements by the first sampling and then extract features for RDO.

The CS measurements with different sampling rates are subsets of the measurement population for the same image, so a small number of measurements can be used to estimate the features of measurements with a higher sampling rate. In this paper, $m_{0}$ represents the sampling rate of the first sampling.

The entropy-matching method is usually used to estimate the shape parameter of the generalized Gaussian distribution [31]. To simplify the estimation, we assume that there is a proportional relationship between $-\log_{2}\left(\beta {\left[\Gamma (3/\beta )\right]}^{\frac{1}{2}}/\Gamma {\left[(1/\beta )\right]}^{\frac{3}{2}}\right)+1/(\beta \ln2)$ at different sampling rates for the same bit-depth, i.e.,

(8) $$\begin{array}{l} -\log_{2}\left(\beta {\left[\Gamma (3/\beta )\right]}^{\frac{1}{2}}/\Gamma {\left[(1/\beta )\right]}^{\frac{3}{2}}\right)+1/(\beta \ln2)\\ \approx c\left(-\log_{2}\left(\beta_{0}{\left[\Gamma \left(3/\beta_{0}\right)\right]}^{\frac{1}{2}}/\Gamma {\left[\left(1/\beta_{0}\right)\right]}^{\frac{3}{2}}\right)+1/\left(\beta_{0}\ln2\right)\right)\\ \approx c\left(H_{0}-\frac{1}{2}\log_{2}\left(2\sigma_{f_{0}}\right)+\log\Delta \right) \end{array}$$

where $H_{0}$ and $\beta_{0}$ represent the information entropy and shape parameter of f(y) at sampling rate $m_{0}$ and bit-depth b. c is an undetermined parameter.

Combined with the Formula (8), the information entropy of $y_{Q}$ can be estimated by the following expression:

(9) $$H\approx \frac{1}{2}\log_{2}\left(2\sigma_{f}\right)+c\left(H_{0}-\frac{1}{2}\log_{2}\left(2\sigma_{f_{0}}\right)+\log\Delta_{f_{0}}\right)-\log\Delta ,$$

where $\sigma_{f_{0}}$ is the standard deviation of f(y) for measurements obtained by the first partial sampling, $\Delta_{f_{0}}=\frac{f_{\max}\left(y_{0}\right)-f_{\min}\left(y_{0}\right)}{2^{b}}$, $y_{0}$ is the measurement vector obtained by the first sampling.

In statistical theory, the statistic $s^{2}=\frac{M}{M-1}\sigma_{}^{2}$ of the sample variance is an unbiased estimation of the population’s variance. Since the CS measurements with different sampling rates have the same population, we assume that the unbiased variance estimates of CS measurements at different measurement rates are approximately equal, that is:

(10) $$\frac{M}{M-1}\sigma_{f}^{2}\approx \frac{M_{0}}{M_{0}-1}\sigma_{f_{0}}^{2},$$

where $M=\mathrm{round}(mN^{2}),M_{0}=\mathrm{round}(m_{0}N^{2})$. The expression (10) can be converted into $\sigma_{f}^{2}\approx \left(\frac{m_{0}N^{2}}{\left(m_{0}N^{2}-1\right)}+\frac{-m_{0}}{\left(m_{0}N^{2}-1\right)}\times \frac{1}{m}\right)\sigma_{f_{0}}^{2}$. Then, we can obtain the following expression:

(11) $$\begin{array}{ll} H & \approx \frac{1}{4}\log_{2}\left(\sigma_{f}^{2}\right)+\frac{1}{2}\log_{2}\left(2\right)+c\left(H_{0}-\frac{1}{2}\log_{2}\left(2\sigma_{f_{0}}\right)+\log\Delta_{f_{0}}\right)-\log\Delta \\ & \approx \frac{1}{4}\log_{2}\left(\frac{m_{0}N^{2}}{\left(m_{0}N^{2}-1\right)}+\frac{-m_{0}}{\left(m_{0}N^{2}-1\right)}\times \frac{1}{m}\right)+\frac{1}{4}\log_{2}(\sigma_{f_{0}}^{2})\\ & \;\;+\frac{1}{2}\log_{2}\left(2\right)+c\left(H_{0}-\frac{1}{2}\log_{2}\left(2\sigma_{f_{0}}\right)+\log\Delta_{f_{0}}\right)-\log\Delta \end{array}$$

Since $\frac{-m_{0}}{\left(m_{0}N^{2}-1\right)}\to \frac{-1}{N^{2}}$ is very small, and the range of the sampling rate m is limited; the range of $\frac{-m_{0}}{\left(m_{0}N^{2}-1\right)}\times \frac{1}{m}$ is also very limited. Therefore, we use a simple linear function to estimate it:

(12) $$\log_{2}\left(\frac{m_{0}N^{2}}{\left(m_{0}N^{2}-1\right)}+\frac{-m_{0}}{\left(m_{0}N^{2}-1\right)}\times \frac{1}{m}\right)\approx c\prime \frac{1}{m}+c\prime \prime ,$$

where c′ and c″ are undetermined parameters. Moreover, we substitute the expression of quantization step into (11) to obtain the following expression:

(13) $$\begin{array}{l} c\log_{2}\Delta_{f_{0}}-\log_{2}\Delta \\ =c\log_{2}\frac{f_{\max}\left(y_{0}\right)-f_{\min}\left(y_{0}\right)}{2^{b}}-\log_{2}\frac{f_{\max}\left(y\right)-f_{\min}\left(y\right)}{2^{b}}\\ =\left(1-c\right)b+c\log_{2}\left(f_{\max}\left(y_{0}\right)-f_{\min}\left(y_{0}\right)\right)-\log_{2}\left(f_{\max}\left(y\right)-f_{\min}\left(y\right)\right) \end{array}$$

We used the maximum $f_{\max}\left(y_{0}\right)$ and minimum $f_{\min}\left(y_{0}\right)$ of the first sampled CS measurements to predict the maximum $f_{\max}\left(y\right)$ and minimum $f_{\min}\left(y\right)$ of the CS measurements with sampling rate m. Therefore,

(14) $$\begin{array}{ll} H\approx & c\prime \frac{1}{m}+c\prime \prime +(\frac{1}{4}-\frac{1}{4}c)\log_{2}\sigma_{f_{0}}^{2}+\log_{2}\left(2\right)\\ & +cH_{0}+\left(1-c\right)b+\left(c-1\right)\log_{2}\left(f_{\max}\left(y_{0}\right)-f_{\min}\left(y_{0}\right)\right) \end{array}$$

Based on (14), we replaced (1−c), c′, c, $\left(\frac{1}{4}-\frac{1}{4}c\right)$, (c−1) and $c\prime \prime +\log_{2}\left(2\right)$ with $c_{1}$, $c_{2}$, $c_{3}$, $c_{4}$, $c_{5}$ and $c_{6}$, respectively, and establish a model for estimating the average codeword length as follows:

(15) $$L\approx c_{1}b+\frac{c_{2}}{m}+c_{3}H_{0}+c_{4}\log_{2}\sigma_{f_{0}}^{2}+c_{5}\log_{2}\left(f_{\max}\left(y_{0}\right)-f_{\min}\left(y_{0}\right)\right)+c_{6}.$$

To improve the estimation accuracy of the average codeword length, we utilize the model coefficients $c_{1}∼c_{6}$ learned from offline data. Combining (3) with (15), we can establish the bit-rate model, as follows:

(16) $$R\approx mL\approx m[c_{1}b+\frac{c_{2}}{m}+c_{3}H_{0}+c_{4}\log_{2}\sigma_{f_{0}}^{2}+c_{5}\log_{2}\left(f_{\max}\left(y_{0}\right)-f_{\min}\left(y_{0}\right)\right)+c_{6}],$$

## 4. Optimal Bit-Depth Model

If we first predict the optimal bit-depth $b^{*}$, the sampling rate can be estimated based on the bit-rate model (16), i.e.,

(17) $$m^{*}\approx \frac{R_{goal}-c_{2}}{c_{1}b^{*}+C},$$

where $R_{goal}$ is the target bit-rate, $C=c_{3}H_{0}+c_{4}\log_{2}\sigma_{f_{0}}^{2}+c_{5}\log_{2}\left(f_{\max}\left(y_{0}\right)-f_{\min}\left(y_{0}\right)\right)+c_{6}$ represents the feature of X at bit-depth $b^{*}$. In this section, we propose an optimal bit-depth model, which can directly predict the optimal bit-depth for a given bit-rate.

### 4.1. Function Mapping Relationship between Optimal Bit-Depth and Bit-Rate

**Figure 3:**
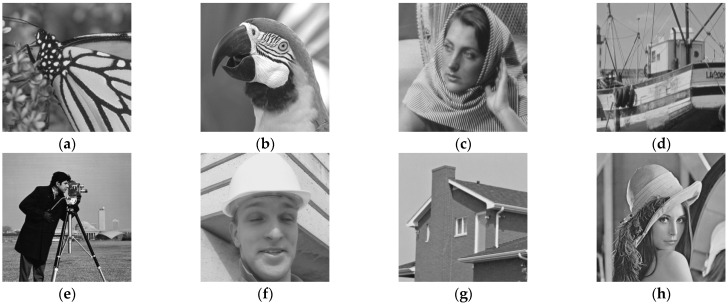
Eight testing images. (**a**) Monarch; (**b**) Parrots; (**c**) Barbara; (**d**) Boats; (**e**) Cameraman; (**f**) Foreman; (**g**) House; (**h**) Lena.

**Figure 4:**
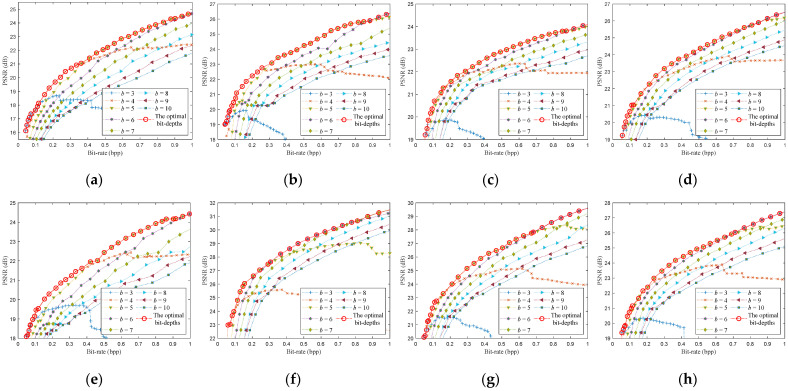
The optimal bit-depths of eight images for CS-based coding system with uniform SQ. (**a**) Monarch; (**b**) Parrots; (**c**) Barbara; (**d**) Boats; (**e**) Cameraman; (**f**) Foreman; (**g**) House; (**h**) Lena.

**Figure 5:**
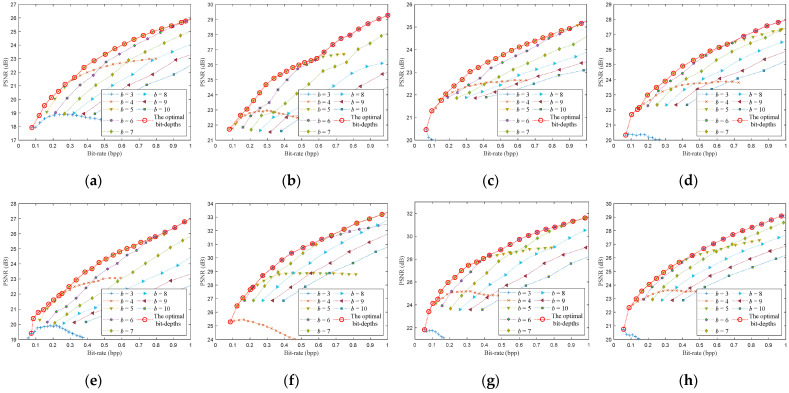
The optimal bit-depths of eight images for CS-based coding system with DPCM-plus-SQ. (**a**) Monarch; (**b**) Parrots; (**c**) Barbara; (**d**) Boats; (**e**) Cameraman; (**f**) Foreman; (**g**) House; (**h**) Lena.

Chen et al. [15] tested the reconstruction performance of some images at different quantization bit-depths. They show that low quantization bit-depths can reconstruct high PSNRs at a low bit-rate, and the high quantization bit-depths can reconstruct high PSNRs at high bit-rate. However, they only give the fixed selection of quantization bit-depths for some bit-rates of all images, and do not give a method for selecting the optimal bit-depth. To find the relationship between the different quantization bit-depths and the PSNRs, we simulated eight test images, as shown in Figure 3. We obtain the optimal bit-depths of eight testing images by traversing different sampling rates (m∈[0.05,0.06,…,0.4]) and different quantization bit-depths (b∈[3,4,…,10]) for CS-based coding systems with uniform SQ and DPCM-plus-SQ, as shown in Figure 4 and Figure 5.

It can be found from Figure 4 and Figure 5 that the rate-distortion performance of the DPCM-plus-SQ framework (represents the CS-based coding system with DPCM-plus-SQ) is better than that of the uniform SQ framework (represents the CS-based coding system with uniform SQ), which indicates that the quantization scheme has a significant influence on the rate-distortion performance. However, the current rate-distortion optimization methods for CS are only suitable for a single uniform SQ framework. As far as we know, little attention has been paid to study the rate-distortion optimization method suitable for the prediction framework.

Although the optimal bit-depth of different quantization frameworks is different, Figure 4 and Figure 5 have the following common characteristics: (1) low bit-depths have high PSNRs at low bit-rates, and high bit-depths have high PSNRs at high bit-rates. (2) The optimal bit-depth of almost all images is 4 when the bit-rate is around 0.1 bpp. (3) With the increase of bit-rate, the optimal bit-depth shows a nondecreasing trend. (4) The optimal bit-depth is the same in a bit-rate range, but the range is different for different images. There is a functional relationship between the optimal bit-depth and the bit-rate, which can be expressed as:

(18) $$b_{best}=\{\begin{array}{c} 3\;\;\;0<R\leq r_{1}\\ \begin{array}{c} 4\;\;r_{1}<R\leq r_{2}\\ \vdots \end{array}\\ 8\;\;r_{5}<R\leq r_{6} \end{array},$$

where $r_{1}∼r_{6}$ are the endpoints of the bit-rate ranges. It can be found that the bit-rate range increases with the increases of $b_{best}$. The model (18) is equivalent to the following model:

(19) $$b_{best}=\left[g(R)\right],$$

where [·] represents the rounding operation, and g(R) represents a continuous function of the bit-rate. Since the optimal bit-depth increases with the increases of bit-rate, the first-order derivative of g(R) is required to be no less than 0. The increasing rate of the optimal bit-depth becomes slower with the increase of bit-rate, so the second-order derivative of g(R) is required to be less than 0, that is,

(20) $$\frac{\partial g{\left(R\right)}^{2}}{\partial^{2}R}<0\;\;\&\;\;\frac{\partial g(R)}{\partial R}\geq 0,$$

Based on the above discussion, we set $g(R)=k_{1}\ln(R)+k_{2}$. The model of the optimal bit-depth is established as follows:

(21) $${\tilde{b}}_{best}=\left[g(R)\right]=\left[k_{1}\ln(R)+k_{2}\right],$$

where $k_{1}$ and $k_{2}$ are the model parameters, which are learned by a neural network in the Section 4.2. In order to collect offline data samples of $k_{1}$ and $k_{2}$ for the proposed neural network training, we establish the following optimization problem:

(22) $$\underset{k_{1},k_{2}}{\arg\min}\;\sum_{i}\omega_{i}{\left\|b_{best}^{\left(i\right)}-\left[g(R^{\left(i\right)})\right]\right\|}_{1}^{q}+\lambda \sum_{i}{\left\|b_{best}^{\left(i\right)}-g(R^{\left(i\right)})\right\|}_{2}^{2},$$

where i is the sample index of the offline data. $b_{best}^{\left(i\right)}$ represents the actual value of the optimal bit depth of the *i*-th sample. $\omega_{i}$ represents the weight, which is the difference between the PSNR quantized with $b_{best}^{\left(i\right)}$ and the PSNR quantized with $\left[g(R^{\left(i\right)})\right]$ at the same bit-rate. In order to obtain the PSNR at the same bit rate, we perform linear interpolation on the sample data. The regularization term $\sum_{i}{\left\|b_{best}^{\left(i\right)}-g(R^{\left(i\right)})\right\|}_{2}^{2}$ guarantees the uniqueness of the solution. λ is a constant coefficient, which takes 0.01 in this work. We take q=10, which avoids an error of more than 2 bits between the predicted value and the actual value.

In (22), the first item ensures the accuracy of the optimal bit-depth model, and the second item ensures the uniqueness of the model coefficient. Since it is difficult to deal with the gradient of the rounding operation, (22) cannot be solved by the traditional gradient-based optimization method. We use the particle swarm optimization algorithm [32,33] to optimize the problem (22). The number of particle swarm is 100 and iterated 300 times. In each iteration, 30 particle swarms in the population are randomly generated in the [−0.5, 0.5] range of the optimal point.

**Figure 6:**
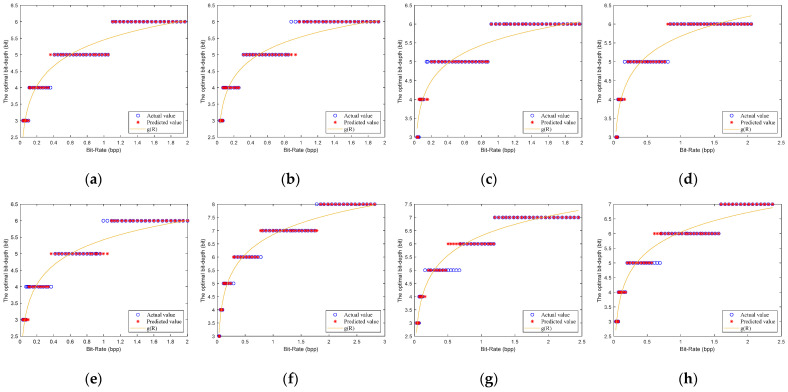
The predicted bit-depths of eight images for the SQ framework. (**a**) Monarch; (**b**) Parrots; (**c**) Barbara; (**d**) Boats; (**e**) Cameraman; (**f**) Foreman; (**g**) House; (**h**) Lena.

**Figure 7:**
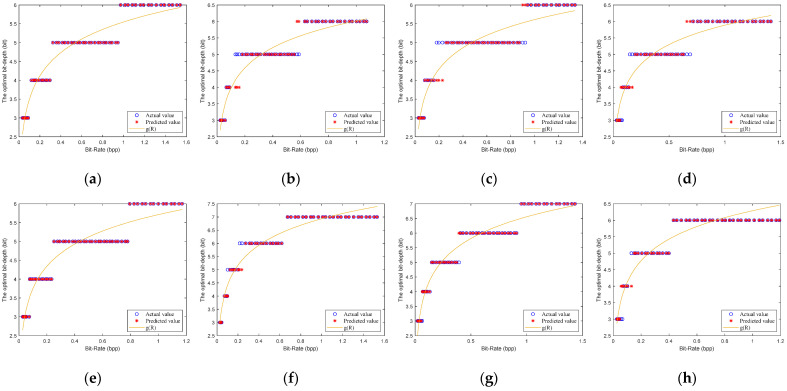
The predicted bit-depths of eight images for the DPCM-plus-SQ framework. (**a**) Monarch; (**b**) Parrots; (**c**) Barbara; (**d**) Boats; (**e**) Cameraman; (**f**) Foreman; (**g**) House; (**h**) Lena.

Figure 6 and Figure 7 show the fitted results of the model (21) for the uniform SQ framework and DPCM-plus-SQ framework, respectively. It can be seen that the fitted bit-depths are in good agreement with the actual bit-depths. The errors between the predicted value and the actual value are only one bit at most. The errors of one bit are mainly concentrated between the two adjacent optimal bit-depths, which has little difference on the PSNR for the two bit-depths.

### 4.2. Model Parameter Estimation Based on Neural Network

It is challenging to design a function for estimating the model parameters accurately. Therefore, we use a four-layer feed-forward neural network [34,35] to learn the mapping relationship between the model parameters and image features rather than designing the function relationship by hand [36,37]. We can imagine that the model (21) would be beneficial if the model parameters could be predicted based on some content features derived from the compressed sampled image. As model (21) is closely related to the bit-rate, we directly use the image features in the proposed bit-rate model as the characteristics of estimating the parameters. The image features of the proposed bit-rate model are $\sigma_{0}^{}{}^{2}$, $H_{0}$,$f_{\max}\left(y_{0}\right)$ $f_{\min}\left(y_{0}\right)$. A finite set of real numbers usually needs to be quantized before calculating the information entropy. The optimal bit-depth of many images is low when the bit-rate is low, so we choose the information entropy $H_{0,bit=4}$ with a quantization bit-depth of 4 as a feature. Since the CS measurement of the image is sampled block by block, we take the image block as the video frame and design two image features according to the video features in reference [23]. For example, block difference (BD): the mean (and standard deviation) of the difference between the measurements of adjacent blocks, i.e., $\mu_{\mathrm{BD}}$ and $\sigma_{\mathrm{BD}}$. We also take the mean of measurements ${\bar{y}}_{0}$ as a feature.

**Figure 8:**
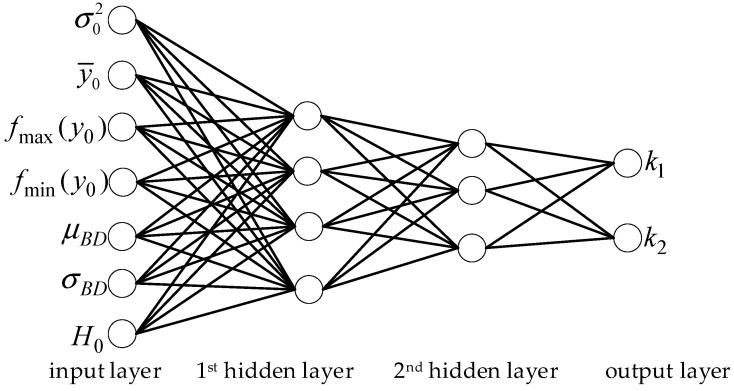
Four-layer feed-forward neural network model for the parameters.

We designed a network including an input layer of seven neurons and an output layer of two neurons to estimate the model parameters $\left[k_{1},k_{2}\right]$, as shown in Formula (23) and Figure 8.

(23) $$\begin{array}{l} u_{1}={\left[\sigma_{0}^{2},{\bar{y}}_{0},f_{\max}\left(y_{0}\right),f_{\min}\left(y_{0}\right),\mu_{BD},\sigma_{BD},H_{0,bit=4}\right]}^{T}\\ u_{j}=g(W_{j-1}u_{j-1}+d_{j-1})\;\;,2\leq j<4\\ F=W_{j-1}u_{j-1}+d_{j-1}\;\;,j=4 \end{array}$$

where g(v)  is the sigmoid activation function, $u_{j}$ is the input variable vector at the *j*-th layer, F is the parameters vector $\left[k_{1},k_{2}\right]$. $W_{j},d_{j}$ are the network parameters learned from offline data. We take the mean square error (MSE) as the loss function.

## 5. A General Rate-Distortion Optimization Method for Sampling Rate and Bit-Depth

### 5.1. Sampling Rate Modification

The model (16) obtains the model parameters by minimizing the mean square error of all training samples. Although the total error is the smallest, there are still some samples with significant errors. To prevent excessive errors in predicting sampling rate, we propose the average codeword length boundary and sampling rate boundary.

#### 5.1.1. Average Codeword Length Boundary

When the optimal bit-depth is determined, the average codeword length usually decreases with the sampling rate increase. Although the average codeword length of different images varies with the sampling rate, the variation is finite. Therefore, we design an average codeword length boundary.

As the information entropy $H_{0}$ is the input of the optimized sampling rate and is very close to the average codeword length $L_{0}$ with the sampling rate $m_{0}$, we take $H_{0}$ as the reference of the average codeword length to estimate variation. The average codeword length variation is expressed as $L-H_{0}$. We only take the bit-depth and sampling rate as factors for influencing the upper and lower bound. According to model (16), we establish the upper and lower bound model of the average codeword length variation as follows:

(24) $$\begin{array}{l} L_{u}-H_{0}=a_{1}b+\frac{a_{2}}{m}+a_{3}\\ L_{l}-H_{0}=a_{4}b+\frac{a_{5}}{m}+a_{6} \end{array}$$

where $L_{u}$ and $L_{l}$ describe the upper and lower bounds of average codeword length, respectively. $a_{1}∼a_{6}$ are the model coefficients fitted by offline samples.

According to (17), we first estimate the sampling rate as

(25) $$m^{\left(1\right)}=(R_{goal}-c_{3})/(c_{1}b^{*}+C)$$

The corresponding average codeword length is $L=R_{goal}/m^{\left(1\right)}$. Then, we calculate the upper $L_{u}=a_{1}b^{*}+a_{2}/m+a_{3}+H_{0}$ and the lower bound $L_{l}=a_{4}b^{*}+a_{5}/m+a_{6}+H_{0}$ based on (24). $L>L_{u}$ means that the sampling rate is too low; we should increase the sampling rate. So, we take the bit-rate model as $R=mL_{u}$, the sampling rate is updated to $m_{u}=(R_{goal}-a_{2})/(H_{0}+a_{1}b^{*}+a_{3})$; if $L<L_{l}$, we take the bit-rate model as $R=mL_{l}$, the sampling rate is updated to $m_{l}=(R_{goal}-a_{5})/(H_{0}+a_{4}b^{*}+a_{6})$. It is summarized as follows:

(26) $$m^{\left(2\right)}=\{\begin{array}{c} m_{u}\;\;\;\;if\;L>L_{u}\\ m_{l}\;\;\;\;\;if\;L<L_{l}\\ m^{\left(1\right)}\;\;otherwise \end{array}$$

#### 5.1.2. Sampling Rate Boundary

The average codeword length boundary uses the information entropy of partial measurements to restrict the estimated value of the average codeword length, so as to modify a sampling rate that is too large or too small. To modify the sampling rate more directly, we establish a linear boundary model of the sampling rate for different bit-depths as follows:

(27) $$\begin{array}{l} m_{u}\prime =a_{7}R+a_{8}\\ m_{l}\prime =a_{9}R+a_{10} \end{array}$$

where R is the bit-rate, $a_{7}∼a_{10}$ are the model coefficients fitted by offline samples. When the assigned sampling rate exceeds the boundaries in (27), it will be modified by the following expression:

(28) $$m^{*}=\{\begin{array}{c} m_{u}\prime \;\;\;\;if\;m^{\left(2\right)}>m_{u}\prime \\ m_{l}\prime \;\;\;\;\;if\;m^{\left(2\right)}<m_{l}\prime \end{array}$$

### 5.2. Rate-Distortion Optimization Algorithm

Based on the proposed bit-rate model and the optimal bit-depth model, we propose an algorithm to assign the bit-depth and sampling rate for a given target bit-rate $R_{goal}$, as follows.

(1) Partial sampling.

The partial CS measurements are sampled with the sampling rate $m_{0}$.

(2) Features extraction.

$\sigma_{0}^{2},{\bar{y}}_{0},f_{\max}\left(y_{0}\right),f_{\min}\left(y_{0}\right),\mu_{BD},\sigma_{BD},H_{0,bit=4}$ of partial measurements are calculated.

(3) The optimal bit-depth prediction.

The optimal bit-depth is predicted by $b_{best}=\left[k_{1}\ln(R)+k_{2}\right]$, where the model parameters are estimated by the trained network.

(4) Features extraction.

The partial measurements are quantized with bit-depth $b^{*}$, and then the information entropy $H_{0}$ is calculated.

(5) The optimal sampling rate prediction.

The optimal sampling rate is estimated by Formula (25).

(6) Sampling rate modification

The sampling rate is updated according to the Formulas (26) and (28).

(7) CS sampling

The original image is acquired to obtain the remaining CS measurements by the supplementary sampling rate $m=m^{*}-m_{0}$.

(8) Quantization and entropy encoding.

All measurements are quantized and then coded by arithmetic coding.

### 5.3. Computational Complexity Analysis

The extra calculation of the sampling rate and quantization bit-depth optimization comes from three processes, namely feature extraction, the optimal bit-depth prediction, and the sampling rate estimation.

In feature extraction, the extra calculation mainly comes from $\sigma_{0}^{2},{\bar{y}}_{0},f_{\max}\left(y_{0}\right),f_{\min}\left(y_{0}\right),\mu_{BD},\sigma_{BD},H_{0,bit=4}$ of the measurements with sampling rate $m_{0}$. The number of measurements is $m_{0}\times N^{2}$. We assume that the calculation of one subtraction is equivalent to that of one addition. The calculation of ${\bar{y}}_{0}$ needs $m_{0}\times N^{2}-1$ additions and one multiplication. The calculation of $\sigma_{0}^{2}$ needs $m_{0}\times N^{2}\times 2-1$ additions and $m_{0}\times N^{2}+1$ multiplications. $f_{\max}\left(y_{0}\right)$ and $f_{\min}\left(y_{0}\right)$ need $\left(m_{0}\times N^{2}-1\right)\times 2$ judgment operations. The calculation of block errors needs $m_{0}\times (N^{2}-B^{2})$ additions. $\mu_{BD}$ needs $m_{0}\times (N^{2}-B^{2})-1$ additions and one multiplication. $\sigma_{BD}$ needs $m_{0}\times (N^{2}-B^{2})\times 2-1$ additions and $m_{0}\times (N^{2}-B^{2})+1$ multiplications. The extra calculation of $H_{0,bit=4}$ comes from quantization with bit-depth 4, which requires $m_{0}\times N^{2}$ multiplications. The remaining calculation of $H_{0,bit=4}$ mainly comes from counting the number of symbols and calculating the entropy. The calculation of counting the number of symbols requires $m_{0}\times N^{2}$ judgments, $m_{0}\times N^{2}$ additions. As the maximum number of symbols is $2^{4+1}$ + 1 = 33, the calculation of entropy needs 66 multiplications, 33 logarithms, and 33 additions at the most.

In the optimal bit-depth prediction, the calculation mainly comes from the neural network model. There are seven neurons in the input layer, two neurons in the output layer, four neurons in the first hidden layer, and three neurons in the second hidden layer. When the activation function is not considered, the calculation of the network includes 7 × 4 + 4 × 3 + 3 × 2 = 46 multiplications and 6 × 4 + 4 + 3 × 3 + 3 + 2 × 2 + 2 = 46 additions. In the sampling rate estimation, the amount of calculation mainly comes from the calculation of (25) and (26).

Compared with the computations of the CS measurements, a fixed number of operations can be ignored. The extra calculation includes $m_{0}\times N^{2}\times 8$ additions, $m_{0}\times N^{2}\times$ 3 multiplications, and $m_{0}\times N^{2}\times$ 3 judgments. Assuming that two additions are needed for one judgment operation, the total amount of additional computation requires $m_{0}\times N^{2}\times 14$ additions and $m_{0}\times N^{2}\times 3$ multiplications.

The computations of all CS measurements requires $m\times N^{2}\times B^{2}$ multiplications and $m\times N^{2}\times (B^{2}-1)$ additions. B is the size of the image block, which is at least 16. When B = 16, the optimization process needs to increase $3/B^{2}\times \left(m_{0}/m\right)\leq 3/256$ ≈1.17% multiplications and $14/{(B}^{2}-1)\times \left(m_{0}/m\right)\leq 14/255\approx 5.49\%$ additions. In computer operations, the amount of calculation of addition is at least ten times faster than multiplication. The computations of rate-distortion optimization will not exceed 2% of the computations of the partial measurements. Furthermore, with the increase of image block size or sampling rate m, the percentage of computation in the optimization process will be further reduced.

## 6. Experimental Results

The proposed method is tested on some images for the DPCM-plus-SQ framework and uniform SQ framework, respectively. The model parameters are obtained by offline training some images of the BSDS500 database [38]. Several images, including eight images (shown in Figure 3), and the BSD68 dataset [39], are tested in our simulations. We take 100 images randomly selected from the BSDS500 database as the training set and the BSD68 dataset (68 images) as the test set. Since the size of the images varies, the images were cropped to a size of 256 × 256 from the center. All the numerical experiments are performed via MATLAB (R2018b) on a Windows 10 (64 bit) platform with an Intel Core i5-8300H 2.30 GHz processor and 16 GB of RAM.

### 6.1. Model Parameters Estimation

To obtain the model parameters of the proposed bit-rate model and the optimal bit-depth model, we take 100 images from the BSDS500 database [38] to collect training samples. The training data adopts the way of traversing bit-depths and sampling rates. The bit-depths include {3, 4, …, 10}; the set of sampling rate includes 37 samples in {0.04, 0.05, …, 0.4} and 7 samples in {3/256, 4/256, …, 9/256}. If the average codeword length compressed by entropy encoding is greater than the quantized bit-depth, we take the average codeword length equal to the quantized bit-depth. One image collects 352 samples of the average codeword length and PSNR. The image block size adopts the optimal size of the corresponding quantization method, in which the DPCM quantization framework uses 16×16 blocks and uniform quantization uses 32 × 32 blocks. The orthogonal random Gaussian matrix is used for BCS sampling in this work. The entropy encoder adopts arithmetic coding [40]. In the decoder, the SPL-DWT algorithm [41] is used for image reconstruction. We take the first partial sampling rate $m_{0}=0.05$.

We use the least-square method to fit the model (15). Table 1 shows the trained parameters for DPCM-plus-SQ framework and uniform SQ framework. To quantify the accuracy of the fitting, we calculate the mean square error (MSE) and Pearson correlation coefficient (PCC) [42] between the actual value and predicted value. The closer the PCC is to 1, the better the fit of the model. The closer the MSE is to 0, the better the fit of the model. For the DPCM-plus-SQ framework, the MSE and PCC are 0.022 and 0.995, respectively. For the uniform SQ framework, the MSE and PCC are 0.027 and 0.996, respectively. Table 1 shows that the proposed model (15) can well describe the relationship between average codeword length L and bit-depth, sampling rate, and image features. The results show that model (15) can well describe the relationship between the average codeword length, sampling rate, and bit-depth.

The optimal bit-depth model depends on the model parameters estimated by the proposed neural network. The samples of the model parameters are obtained by solving the problem (22) and then are used to train the neural network. Due to the random initialization of neural network parameters, the prediction performances of the different trained networks are different. The best network from several trained networks is chosen to estimate the parameters of the proposed optimal bit-depth model. Table 2 shows the prediction performance of the optimal bit-depth model in the training set image and test set.

As shown in Table 2, for DPCM-plus-SQ framework, 80.7% and 70.7% are the accuracies of predicting the optimal bit-depth in the training set (BSDS500) and the test set (BSD68), respectively. For uniform SQ framework, 76.5% and 70.4% are the accuracies of predicting the optimal bit-depth in the training set (BSDS500) and the test set (BSD68), respectively. In the training set, the differences between the optimal bit-depth and the predicted bit-depth are no more than one bit. In the test set, 99.7% of the samples have a difference of no more than one bit between the optimal bit-depth and the predicted bit-depth. In most cases, the influence of 1-bit error on PSNR is limited, so it is effective to utilize a neural network to learn the optimal bit-depth model parameters.

In the training set, 100 images have 100 the average codeword length curves. We take the upper five curves and the lower five curves as the training samples of the model (24). The parameters are fitted offline by the least square method. We obtain $a_{1}$ = $-8.4564\times 10^{-3}$, $a_{2}$ = $1.8272\times 10^{-2}$, $a_{3}$ = $-1.5871\times 10^{-1}$, $a_{4}$ = $-6.7478\times 10^{-2}$, $a_{5}$ = $1.4306\times 10^{-2}$, $a_{6}$ = $-1.8052\times 10^{-1}$ for the DPCM-plus-SQ framework, and $a_{1}$ = $4.5857\times 10^{-2}$, $a_{2}$ = $8.1957\times 10^{-3}$, $a_{3}$ = $-2.1012\times 10^{-1}$, $a_{4}$ = $1.0226\times 10^{-1}$, $a_{5}$ = $1.6633\times 10^{-3}$, $a_{6}$ = $-9.0052\times 10^{-1}$ for the uniform SQ framework.

In the training set, we take the maximum and minimum sampling rate corresponding to the given bit-rates as the training sample of the model (27). The parameters are obtained by the least-square method. Through experiments, we found that the optimized sampling rates beyond the boundary are mainly near the low bit-rate of 0.1–0.3, and the corresponding optimal bit-depths are mostly 4 bit or 5 bit. So, we impose boundary constraints on the sampling rates when the optimal bit-depths are 4 and 5. The parameters are fitted offline by the least square method. For DPCM-plus-SQ framework with bit-depth of 4, we obtain $a_{7}=4.9164\times 10^{-1}$, $a_{8}=-7.1258\times 10^{-3}$. For DPCM-plus-SQ framework with bit-depth 5, we obtain $a_{7}$ = $3.4874\times 10^{-1}$, $a_{8}=-6.1371\times 10^{-3}$. For uniform SQ framework with bit-depth 4, we obtain $a_{7}=3.3181\times 10^{-1}$, $a_{8}=-1.3050\times 10^{-3}$. For uniform SQ framework with bit-depth 5, we obtain $a_{7}=2.3433\times 10^{-1}$, $a_{8}=2.3347\times 10^{-3}$.

### 6.2. Rate-Distortion Optimization Performance

To verify the accuracy of the bit-rate model, we tested the BSD68 dataset and eight images in Figure 3, respectively. We first use the proposed algorithm to assign the sampling rate and bit-depth for target bit-rates, including 0.1, … 1 bit per pixel (bpp). Then, we calculate actual bit-rates and PSNRs of the reconstructed image for the estimated sampling rate and bit-depth. Table 3 and Table 4 show the optimized bit-rate of BSD68 for the uniform SQ framework and DPCM-plus-SQ framework, respectively. The absolute error percentage denotes the percentage of the absolute error in the target bit-rate, where the absolute error is the absolute of the difference between target bit-rate and actual bit-rate.

As shown in Table 3, the bit-rate average absolute error percentages of BSD68 are between 1.65% and 3.23%, which indicates that the proposed bit-rate model is useful for uniform SQ. As shown in Table 4, the bit-rate average absolute error percentages of BSD68 are between 2.09% and 3.17%, which indicates that the proposed bit-rate model is useful for DPCM-plus-SQ.

Table 5 and Table 6 show the actual bit-rate of the eight testing images for uniform SQ and DPCM-plus-SQ, respectively. The results exhibit that actual bit-rates are very close to the target bit-rates.

To test the validity of the optimal bit-depth model, we compare the predicted optimal bit-depth with the best bit-depths by traversing different bit-depths and different sampling rates, as shown in Table 7 and Table 8.

In Table 7 and Table 8, the optimal percentage shows the percentage of images whose predicted bit-depth is consistent with the actual best bit-depth. The one-bit error percentage is the percentage of images with the one-bit error between the predicted bit-depth and the actual best bit-depth. We encode and decode the images according to the predicted parameters (sampling rate and bit-depth) and calculate the bit-rate and PSNR. The PSNR error is the PSNR minus the maximum PSNR, where the PSNRs are obtained by the nearest interpolation method for a bit-rate.

In Table 7, the sum of optimal bit-depth and one-bit error bit-depth obtain a percentage of between 98.53% and 100% for the SQ framework. When the target bit-rates are 0.1~0.8 bpp, the sum of the optimal bit-depth and one-bit error bit-depth percentage is 100%. When the target bit-rates are 0.9 and 1 bpp, the sum of optimal bit-depth and one-bit error bit-depth percentage is 98.53%. As the difference of PSNR between different bit-depths is small at the high target bit-rates, there is an error in estimating the bit-depth. Although only 54.41% to 91.18% of the predicted bit-depths are consistent with the optimal bit-depth, the average PSNR errors are between 0.04 dB and 0.013 dB, which shows that the error of predicted bit-depth has little influence on the reconstruction performance.

In Table 8, the sum of optimal bit-depth and one-bit error bit-depth obtain a percentage of between 98.53% and 100% for the DPCM-plus-SQ framework. When the target bit-rates are 0.1~0.7 bpp, the sum of optimal bit-depth and one-bit error bit-depth obtain a percentage of 100%. When the target bit-rates are 0.8~1 bpp, the sum of optimal bit-depth and one-bit error bit-depth obtains a percentage of 98.53%. Although only 55.88% to 82.35% of the predicted bit-depths are consistent with the optimal bit-depth, the average PSNR errors are between 0.07 dB and 0.029 dB, which shows that the error of predicted bit-depth has little influence on the reconstruction performance.

**Figure 9:**
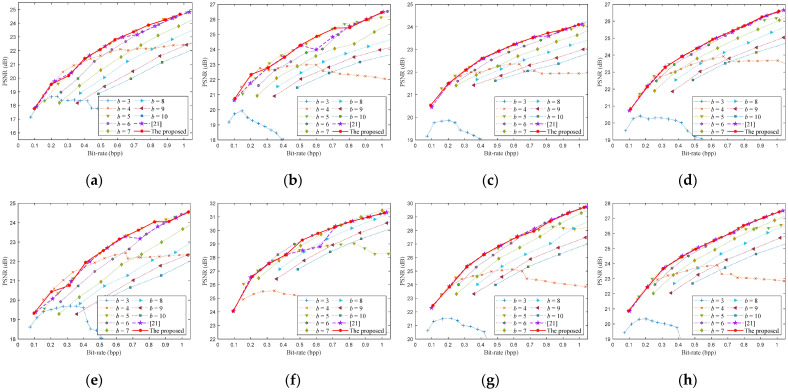
Rate-distortion performance of the proposed algorithm for the SQ framework. (**a**) Monarch; (**b**) Parrots; (**c**) Barbara; (**d**) Boats; (**e**) Cameraman; (**f**) Foreman; (**g**) House; (**h**) Lena.

**Figure 10:**
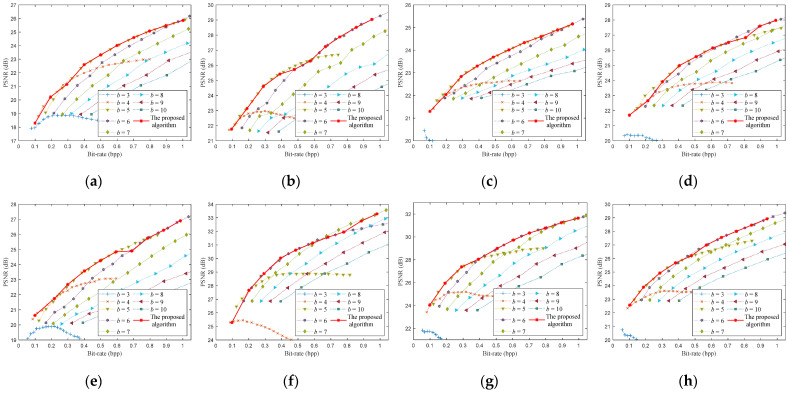
Rate-distortion performance of the proposed algorithm for the DPCM-plus-SQ framework. (**a**) Monarch; (**b**) Parrots; (**c**) Barbara; (**d**) Boats; (**e**) Cameraman; (**f**) Foreman; (**g**) House; (**h**) Lena.

To demonstrate the performance of the proposed method in detail, we give the optimized rate-distortion curves of the eight testing images, as shown in Figure 9 and Figure 10. We first encode the image for the bit-rates according to the optimized sampling rates and bit-depths, then calculate the PSNRs of the reconstructed image to obtain the rate-distortion curve. All bit-rates and PSNRs obtained by traversing different sampling rates and bit-depths are also shown in Figure 9 and Figure 10. As far as we know, the optimization of sampling rate and bit-depth in the CS-based coding system is mainly focused on the uniform SQ framework, so we compared the proposed method with the latest methods [21] for the uniform SQ framework, as shown in Figure 9.

Figure 9 shows the proposed algorithm’s rate-distortion curves of the eight test images encoded by the CS-based coding system with uniform SQ. The rate-distortion curve of the proposed algorithm is very close to the optimal rate-distortion curve. The PSNRs of the proposed algorithm are slightly worse than the optimal PSNRs only at a few bit-rates. When the bit-rate is 0.3 bpp for Monarch, the predicted optimal bit-depth is 5 bit, while the actual optimal bit-depth is 4 bit. The PSNR of the proposed algorithm is 0.52 dB less than the optimal PSNR at bit-rate of 0.3 bpp. When the bit-rate is 0.3 and 0.8 bpp for Parrots, the predicted optimal bit-depth is 4 and 6, while the actual optimal bit-depth is 5 bit. The PSNRs of the proposed algorithm are about 0.25 and 0.3 dB less than the optimal PSNR at bit-rate of 0.3 and 0.8 bpp. When the bit-rate is 0.3 and 0.9 bpp for Cameraman, the predicted optimal bit-depth is 5 and 6, while the actual optimal bit-depth is 4 and 5. The PSNRs of the proposed algorithm are about 0.48 and 0.12 dB less than the optimal PSNR at bit-rate of 0.3 and 0.9 bpp. When the bit-rate is 0.4 bpp for Foreman, the predicted optimal bit-depth is 5 bit, while the actual optimal bit-depth is 6 bit. The PSNR of the proposed algorithm is 0.41 dB less than the optimal PSNR at bit-rate of 0.4 bpp. The optimal bit-depth model mainly causes these deviations, whereas the maximum deviation is 1 bit.

The proposed algorithm’s rate-distortion curves are very close to the results of [21] on Barbara, Boats, House, Lena, and are better than [21] on Monarch, Parrots, Cameraman, Foreman. It can be seen from Figure 9a that the optimal bit-depth is 7 at the bit-rate of 0.7 bpp or 0.8 bpp, the proposed algorithm can accurately predict the optimal bit-depth. However, the bit-depth predicted by [21] is 6, which is one bit less than the optimal bit-depth. In Figure 9b, the optimal bit-depth is 4 at the bit-rate of 0.2 bbp, and the optimal bit-depth is 5 at the bit-rate of 0.6 bpp and 0.7 bbp. Compared with [21], the predicted bit-depths of the proposed algorithm are more accurate. Some similar situations occur in Figure 9e,f.

Figure 10 shows the proposed algorithm’s rate-distortion curves of the eight test images encoded by the CS-based coding system with DPCM-plus-SQ. The rate-distortion curve of the proposed algorithm is very close to the optimal rate-distortion curve. The PSNRs of the proposed algorithm are slightly worse than the optimal PSNRs only at a few bit-rates. When the bit-rate is 0.5 bpp for Parrots, the predicted optimal bit-depth is 6 bit, while the actual optimal bit-depth is 5 bit. The PSNR of the proposed algorithm is 0.25 dB less than the optimal PSNR at bit-rate of 0.5 bpp. When the bit-rate is 0.2 and 0.8 bpp for the image boats, the predicted optimal bit-depth is 4 and 5 bit, while the actual optimal bit-depth is 5 and 6. The PSNRs of the proposed algorithm are about 0.5 dB and 0.4 dB less than the optimal PSNR at bit-rates of 0.2 and 0.8 bpp. When the bit-rate is 0.7 bpp for Cameraman, the predicted optimal bit-depth is 6 bit, while the actual optimal bit-depth is 5 bit. The PSNR of the proposed algorithm is 0.45 dB less than the optimal PSNR at bit-rate of 0.7 bpp. When the bit-rate is 0.8 bpp for Foreman, the predicted optimal bit-depth is 6 bit, while the actual optimal bit-depth is 7 bit. The PSNR of the proposed algorithm is about 0.29 dB less than the optimal PSNR at bit-rate of 0.8 bpp.

From Figure 9 and Figure 10, the prediction deviation of the optimal bit-depth is at most 1 bit, which mainly occurs at the junction between the two optimal bit-depths and has little effect on PSNR. The rate-distortion curves of the proposed algorithm are almost the optimal curve for the DPCM-plus-SQ framework and SQ framework. Although the proposed algorithm’s rate-distortion performance is not optimal at some bit-rates, the gap is small.

## 7. Conclusions

The CS-based coding system needs to assign sampling rate and quantization bit-depth for a given bit-rate before encoding an image. In this work, we first propose a bit-rate model and an optimal bit-depth model for the CS-based coding system. The proposed bit-rate model and optimal bit-depth model have simple mathematical forms, and they have effective parameters based on training off-line data. Then, we propose a general rate-distortion optimization method to assign sampling rate and quantization bit-depth based on the bit-rate model and optimal bit-depth model. The proposed method only needs to extract some features of a small number of measurements, so the computational cost is low. Compared with the first sampling calculation of the CS measurements (blocks’ size is 16×16), the addition and multiplication of the optimization process are about 5.94% and 1.17% of the sampling process, respectively, and the percentage decrease as the block size increases. The disadvantage of the proposed method is that a large amount of offline data needs to be collected to train the model parameters, which is usually acceptable. We test the uniform SQ framework and DPCM-plus-SQ framework, respectively. Experimental results show that the optimized rate-distortion performance and bit-rate of the proposed algorithm are very close to the optimal rate-distortion performance and the target bit-rate.

## Figures and Tables

**Table 1:** Parameters of the fitted model (15).

Quantization Framework	$c_{1}$	$c_{2}$	$c_{3}$	$c_{4}$	$c_{5}$	$c_{6}$	PCC	MSE
DPCM-plus-SQ	−3.0927 × 10^−1^	1.9128 × 10^−2^	−1.6845 × 10^−1^	1.6592 × 10^−1^	1.3467	−1.1718	0.995	0.022
uniform SQ	−2.0660 × 10^−1^	6.5594 × 10^−3^	−2.0673 × 10^−1^	2.3831 × 10^−1^	1.2761	−1.9910	0.996	0.027

**Table 2:** Performances of the training set and test set for the optimal bit-depth model.

Quantization Framework	DPCM-Plus-SQ		Uniform SQ	
Data	Training Set	Test Set	Training Set	Test Set
Accuracy (%)	80.7	70.7	76.5	70.4
Percentage of one-bit error (%)	19.3	29.0	23.5	29.3
Sum (above) (%)	100	99.7	100	99.7

**Table 3:** Comparison of actual bit-rates with target bit-rates for the uniform SQ framework.

**Image**	**Target Bit-Rate (bpp)**		**0.1**	**0.2**	**0.3**	**0.4**	**0.5**
BSD68 test set	Actual bit-rate	Maximum	0.110	0.218	0.327	0.427	0.523
		Minimum	0.087	0.181	0.268	0.368	0.467
		Average	0.099	0.203	0.305	0.406	0.503
	Average of absolute error percentage (%)		3.23	2.91	2.29	2.33	1.78
**Image**	**Target Bit-Rate (bpp)**		**0.6**	**0.7**	**0.8**	**0.9**	**1**
BSD68 test set	Actual bit-rate	Maximum	0.619	0.727	0.831	0.934	1.037
		Minimum	0.565	0.664	0.771	0.831	0.916
		Average	0.603	0.706	0.805	0.901	0.999
	Average of absolute error percentage (%)		1.67	1.87	1.65	1.86	1.86

**Table 4:** Comparison of actual bit-rates with target bit-rates for the DPCM-plus-SQ framework.

**Image**	**Target Bit-Rate (bpp)**		**0.1**	**0.2**	**0.3**	**0.4**	**0.5**
BSD68 test set	Actual bit-rate	Maximum	0.108	0.219	0.319	0.424	0.533
		Minimum	0.090	0.187	0.274	0.366	0.457
		Average	0.101	0.200	0.299	0.399	0.499
	Average of absolute error percentage (%)		3.17	2.75	2.30	2.09	2.03
**Image**	**Target Bit-Rate (bpp)**		**0.6**	**0.7**	**0.8**	**0.9**	**1**
BSD68 test set	Actual bit-rate	Maximum	0.636	0.741	0.848	0.954	1.063
		Minimum	0.548	0.646	0.737	0.829	0.922
		Average	0.597	0.695	0.793	0.891	0.989
	Average of absolute error percentage (%)		2.18	2.23	2.20	2.33	2.26

**Table 5:** The actual bit-rates of eight images for the uniform SQ framework.

Target Bit-Rate (bpp)	Actual Bit-Rate (bpp)							
	Monarch	Parrots	Barbara	Boats	Cameraman	Foreman	House	Lena
0.1	0.102	0.102	0.098	0.106	0.101	0.096	0.105	0.100
0.2	0.203	0.207	0.204	0.207	0.204	0.208	0.204	0.205
0.3	0.305	0.305	0.307	0.317	0.312	0.308	0.310	0.307
0.4	0.408	0.402	0.412	0.416	0.419	0.415	0.414	0.412
0.5	0.493	0.506	0.504	0.513	0.518	0.512	0.511	0.507
0.6	0.590	0.606	0.606	0.615	0.623	0.609	0.612	0.609
0.7	0.694	0.713	0.713	0.725	0.731	0.712	0.720	0.705
0.8	0.790	0.800	0.802	0.827	0.837	0.812	0.817	0.805
0.9	0.884	0.905	0.905	0.918	0.897	0.917	0.923	0.910
1.0	0.981	1.003	1.003	1.019	1.030	1.017	1.024	1.007

**Table 6:** The actual bit-rates of eight images for the DPCM-plus-SQ framework.

Target Bit-Rate (bpp)	Actual Bit-Rate (bpp)							
	Monarch	Parrots	Barbara	Boats	Cameraman	Foreman	House	Lena
0.1	0.102	0.098	0.099	0.103	0.101	0.095	0.098	0.102
0.2	0.197	0.189	0.201	0.210	0.201	0.202	0.190	0.187
0.3	0.303	0.292	0.295	0.308	0.302	0.296	0.291	0.289
0.4	0.404	0.392	0.387	0.410	0.404	0.398	0.407	0.380
0.5	0.504	0.487	0.490	0.511	0.500	0.494	0.505	0.477
0.6	0.603	0.578	0.593	0.611	0.596	0.598	0.610	0.569
0.7	0.705	0.671	0.691	0.703	0.695	0.706	0.703	0.668
0.8	0.799	0.756	0.779	0.811	0.794	0.792	0.804	0.759
0.9	0.899	0.864	0.881	0.902	0.896	0.895	0.898	0.848
1.0	1.014	0.957	0.969	0.993	0.989	0.996	1.004	0.940

**Table 7:** Performance of the optimal bit-depth model for the uniform SQ framework.

**Image**	**Target Bit-Rate (bpp)**	**0.1**	**0.2**	**0.3**	**0.4**	**0.5**
BSD68 test set	Optimal percentage (%)	91.18	64.71	77.94	77.94	64.71
	One-bit error percentage (%)	8.82	35.29	22.06	22.06	35.29
	Sum of the above (%)	100	100	100	100	100
	Average PSNR error (dB)	−0.04	−0.13	−0.12	−0.06	−0.08
**Image**	**Target Bit-Rate (bpp)**	**0.6**	**0.7**	**0.8**	**0.9**	**1**
BSD68 test set	Optimal percentage (%)	63.24	54.41	60.29	57.35	64.71
	One-bit error percentage (%)	36.76	45.59	39.71	41.18	33.82
	Sum of the above (%)	100	100	100	98.53	98.53
	Average PSNR error (dB)	−0.09	−0.10	−0.08	−0.11	−0.07

**Table 8:** Performance of the optimal bit-depth model for the DPCM-plus-SQ framework.

**Image**	**Target Bit-Rate (bpp)**	**0.1**	**0.2**	**0.3**	**0.4**	**0.5**
BSD68 test set	Optimal percentage (%)	82.35	58.82	79.41	79.41	76.47
	One-bit error percentage (%)	17.65	41.18	20.59	20.59	23.53
	Sum of the above (%)	100	100	100	100	100
	Average PSNR error (dB)	−0.29	−0.21	−0.16	−0.06	−0.07
**Image**	**Target Bit-Rate (bpp)**	**0.6**	**0.7**	**0.8**	**0.9**	**1**
BSD68 test set	Optimal percentage (%)	64.71	70.59	60.29	55.88	63.24
	One-bit error percentage (%)	35.29	29.41	38.24	42.65	35.29
	Sum of the above (%)	100	100	98.53	98.53	98.53
	Average PSNR error (dB)	−0.07	−0.09	−0.11	−0.14	−0.11

## Data Availability

Not applicable.
